# Significant Increase in Excess Deaths after Repeated COVID-19 Vaccination in Japan

**DOI:** 10.31662/jmaj.2024-0298

**Published:** 2025-03-07

**Authors:** Hideki Kakeya, Takeshi Nitta, Yukari Kamijima, Takayuki Miyazawa

**Affiliations:** 1 Institute of Systems and Information Engineering, University of Tsukuba, Tsukuba, Japan; 2 Research Institute for Biomedical Sciences, Tokyo University of Science, Noda, Japan; 3 Faculty of Pharmaceutical Sciences, Tokyo University of Science, Noda, Japan; 4 Kyoto Animal Human Organism Research Institute, Kyoto, Japan

**Keywords:** excess death, SARS-CoV-2, chronic infection, adverse reactions, mRNA vaccine, immunosuppression

## Abstract

Although Japan recorded the world’s highest rate of COVID-19 messenger ribonucleic acid (mRNA) vaccination doses per capita, COVID-19 cases and deaths exploded after the emergence of the Omicron variant, followed by a significant increase in excess deaths in 2022 and 2023. Although several hypotheses have been proposed to explain these phenomena, the truth remains to be established because sufficient studies and data disclosures have not been conducted to adequately investigate the possible contribution of mRNA vaccines. The causes of the excess deaths from not only COVID-19 but also other factors after repeated mRNA vaccinations must be elucidated, given this could provide valuable information to help combat future infectious disease outbreaks.

Japan recorded one of the world’s highest rates of COVID-19 vaccination doses per capita, amounting to 3.6 doses as of March 2024 ^(1)^, behind only Cuba and Chile. Because Cuba primarily used protein subunit vaccines ^(2)^ and Chile primarily used inactivated vaccines ^(3)^, Japan has the highest per capita rate of messenger ribonucleic acid (mRNA) vaccination doses in the world.

Japan was regarded as one of the most successful countries in handling the early stages of the pandemic, with much lower numbers of COVID-19 cases and deaths than other developed countries. After the emergence of the Omicron variant, however, the number of infections surged dramatically in Japan in 2022, despite more than 80% of the population having been fully vaccinated. Surprisingly, the number of excess deaths per million in Japan exceeded 1400 in 2023, three times higher than that in the United States, whereas COVID-19 deaths in Japan accounted for only 10% of these excess deaths ^(4)^.

Several hypotheses have been proposed to explain the cause of the significant number of excess deaths in 2022 and 2023. The most popular hypothesis is COVID-19-related deaths, including 1) people who died from COVID-19 but were either not tested or did not receive positive test results and 2) people who died because of the shortage of medical resources due to the surge in COVID-19 cases. On May 8, 2023, however, Japan downgraded COVID-19 from its category as a novel influenza, which required patients with COVID-19 to be treated only at designated medical institutions, to class 5 (the same as seasonal flu), which made it easier for hospitals to treat both patients with and those without COVID-19. Despite this major policy shift, the number of excess deaths in 2023 remained as high as in 2022.

Another hypothesized cause of the excess deaths is various adverse reactions to COVID-19 vaccinations. Indeed, under its relief system for injury to health with vaccination, the government has provided payouts for as many as 8432 injuries including 903 deaths after COVID-19 vaccination as of November 18, 2024 ^(5)^, numbers that are still increasing and already greatly exceed the numbers of injuries and deaths for which payments were made after all other vaccinations in the last 47 years. The aforementioned cases comprise many injuries and deaths in the young population, including the fatal case of a 14-year-old girl ^(6)^.

It is widely supported that vaccination reduced severe illness from COVID-19 in older adults in the early stages of the pandemic, but younger people with low risk of severity were also encouraged to be vaccinated to protect not only themselves but also the older population in Japan. This policy contradicts an early study showing vaccination did not reduce the viral load of infected individuals, which was uploaded to a preprint server in August 2021 but took approximately a year to be published in a journal ^(7)^.

Various adverse reactions to COVID-19 mRNA vaccination have been reported, such as myocarditis, pericarditis, blood clotting, and autoimmune diseases linked to lipid nanoparticles (LNPs) and excessive production of spike proteins generated by the mRNA. Indeed, data on excess deaths in the United Kingdom show that deaths caused by cardiovascular disease increased whereas deaths caused by respiratory disease decreased after COVID-19 vaccination ^(8)^. It is also noteworthy that deaths from cancers related to estrogen receptors, such as leukemia, breast, pancreatic, lip/oral/pharyngeal, ovarian, and uterine cancers, have also increased since the population-wide administration of mRNA vaccinations ^(4)^. The spike protein of SARS-CoV-2 is known to bind to estrogen receptors ^(9)^ located in the nucleus and includes a nuclear localization signal ^(10)^, which makes it more likely to be conveyed to the nucleus.

Another hypothesis involves chronic infection caused by immunosuppression after repeated vaccination. Although adverse reactions were more severe and autoimmune diseases reported more frequently after the second vaccination than the first, they were reported much less after boosters, which can be explained by the suppression of immunity against the SARS-CoV-2 spike protein. Indeed, recent studies have reported an increase in spike-specific immunoglobulin G4, an immunosuppressive class of antibody, and regulatory T cells after the second and subsequent vaccinations ^(11), (12)^. This can lead to chronic infection whereby the virus remains in the intestine, for which positive test results cannot be obtained by nasal swabs. Wastewater monitoring data support this claim. This hypothesis can explain not only the high number and non-COVID-19 ratio of excess deaths but also the synchronization of the COVID-19 infection surge and non-COVID-19 excess deaths.

Former Centers for Disease Control director Robert Redfield said in an interview with former CNN anchor Chris Cuomo ^(13)^, “I believe vaccine has saved many, many people’s lives.” He continued:

“That said, the people’s lives it saved were vulnerable people. People over 60, 65, 70, 75, nursing home people. So, the benefit to them, I think, outweighs the risk.”

“The benefit to the 30-year-old firefighter, I don’t see the benefit.”

He also said:

“There’s prolonged production or impact or negative consequence from spike protein in some people that get the mRNA vaccine.”

Although the truth is yet to be established, the concerns related to the mRNA-LNP formulation evidently need to be taken seriously. Thus, it is imperative to elucidate the effects of population-wide COVID-19 vaccination. Japanese health authorities have been hesitant to provide data since being accused of data mishandling given they classified people vaccinated without recorded dates of inoculation as unvaccinated ^(14)^. On correction, it was revealed that the vaccinated were as susceptible as or even more susceptible to COVID-19 infection than were the unvaccinated ^(15)^.

Given the Japanese population’s uniquely high variation in COVID-19 vaccination numbers, with some having received zero doses and others receiving their eighth from October 2024 onward, data transparency and large-scale research on deaths, injuries, and chronic diseases after COVID-19 vaccination or infection after vaccination can provide valuable insights into the effects of repeated mRNA vaccination, which could greatly aid the world in the fight against future infectious disease outbreaks.

## Article Information

### Conflicts of Interest

None

### Acknowledgement

The authors thank Dr. Yumi Watanabe (Niigata University, Japan) for critical readings of the manuscript and Mr. William Brooks for English proofreading the manuscript.

### Author Contributions

H.K. wrote the manuscript. T.N. supervised the manuscript from an immunological point of view. Y.K. supervised the manuscript from an epidemiological point of view. T.M. supervised the manuscript from a virological point of view.
